# Integrating Artificial Intelligence into Orthodontic Education: A Systematic Review and Meta-Analysis of Clinical Teaching Application

**DOI:** 10.3390/jcm14155487

**Published:** 2025-08-04

**Authors:** Carlos M. Ardila, Eliana Pineda-Vélez, Anny Marcela Vivares Builes

**Affiliations:** 1 Department of Basic Sciences, Saveetha Dental College and Hospitals, Saveetha Institute of Medical and Technical Sciences, Saveetha University, Saveetha 600077, India; 2 Biomedical Stomatology Research Group, Basic Sciences Department, Universidad de Antioquia UdeA, Medellín 050010, Colombia; eliana.pineda@uam.edu.co (E.P.-V.); anny.vivares@uam.edu.co (A.M.V.B.); 3 School of Dentistry, Fundación Universitaria Visión de Las Américas, Medellín 050054, Colombia

**Keywords:** orthodontics, artificial intelligence, dental education, machine learning, cephalometry, stakeholder perception

## Abstract

**Background/Objectives**: Artificial intelligence (AI) is rapidly emerging as a transformative force in healthcare education, including orthodontics. This systematic review and meta-analysis aimed to evaluate the integration of AI into orthodontic training programs, focusing on its effectiveness in improving diagnostic accuracy, learner engagement, and the perceived quality of AI-generated educational content. **Materials and Methods**: A comprehensive literature search was conducted across PubMed, Scopus, Web of Science, and Embase through May 2025. Eligible studies involved AI-assisted educational interventions in orthodontics. A mixed-methods approach was applied, combining meta-analysis and narrative synthesis based on data availability and consistency. **Results**: Seven studies involving 1101 participants—including orthodontic students, clinicians, faculty, and program directors—were included. AI tools ranged from cephalometric landmarking platforms to ChatGPT-based learning modules. A fixed-effects meta-analysis using two studies yielded a pooled Global Quality Scale (GQS) score of 3.69 (95% CI: 3.58–3.80), indicating moderate perceived quality of AI-generated content (I^2^ = 64.5%). Due to methodological heterogeneity and limited statistical reporting in most studies, a narrative synthesis was used to summarize additional outcomes. AI tools enhanced diagnostic skills, learner autonomy, and perceived satisfaction, particularly among students and junior faculty. However, barriers such as limited curricular integration, lack of training, and faculty skepticism were recurrent. **Conclusions**: AI technologies, especially ChatGPT and digital cephalometry tools, show promise in orthodontic education. While learners demonstrate high acceptance, full integration is hindered by institutional and perceptual challenges. Strategic curricular reforms and targeted faculty development are needed to optimize AI adoption in clinical training.

## 1. Introduction

The advent of artificial intelligence (AI) has transformed the landscape of healthcare delivery, with dentistry—and orthodontics in particular—benefiting from novel implementations of machine learning, neural networks, and large language models. AI, defined as the ability of computer systems to mimic and enhance human cognitive processes, has seen exponential growth in daily applications and healthcare technologies [1,2]. AI-driven applications have demonstrated utility in cephalometric analysis, diagnosis, treatment planning, patient triaging, and increasingly, educational enhancement. These systems are not merely supplementary aids for clinical decisions; they are now reshaping how orthodontic students learn and how educators design instructional methods [2].

In orthodontics, AI has been employed for a range of diagnostic and planning tasks, including cervical vertebra maturation analysis, extraction decision-making, orthognathic surgery planning, facial and skeletal age assessment, airway evaluation, and smile simulation using post-treatment imaging software [3,4,5]. This breadth of applications is enabled by advancements in machine learning (ML) and deep learning (DL), particularly through convolutional neural networks (CNNs) and artificial neural networks (ANNs) [5,6].

Multiple studies have evaluated the awareness, perception, and readiness of orthodontic students and professionals to engage with AI-based tools. Gupta et al. [7] reported that although orthodontic postgraduates and faculty members express favorable attitudes toward AI integration, many lack hands-on training and express concerns regarding the ethical and practical limitations of these technologies [6,8,9]. Lin et al. [10] demonstrated that digital cephalometric platforms enhanced landmarking accuracy among preclinical orthodontic students through real-time feedback and visual correction mechanisms. Meanwhile, Metin & Goymen [11] compared AI and human performance in diagnostic interpretation, suggesting that AI models perform comparably or better in structured educational tasks. These findings suggest that AI holds transformative potential for orthodontic education, especially in developing diagnostic acumen and analytical thinking [12].

However, challenges persist. Surveys show that few academic programs have formally integrated AI training into their orthodontic curricula, often due to lack of time, expertise, and institutional guidelines [3,13]. In North America, only about half of accredited orthodontic residency programs report using AI in some capacity, with research applications outpacing clinical or educational use [14]. Similarly, in India, despite strong interest in AI, postgraduate students and clinicians report low usage in tasks like cephalometric analysis, highlighting a disconnect between awareness and actual implementation [5].

Despite these advances, the current body of literature remains fragmented. While individual studies demonstrate promising outcomes, there has yet to be a comprehensive synthesis focused specifically on the educational utility of AI in orthodontics. Most reviews focus on diagnostic accuracy or AI in clinical workflows, leaving a critical gap in our understanding of how AI impacts the cognitive, technical, and professional development of orthodontic learners. As orthodontic curricula evolve, academic institutions face growing pressure to incorporate AI literacy into clinical training and theoretical instruction. Calls have been made to include foundational knowledge about AI principles, capabilities, limitations, and ethical concerns as core components of orthodontic education [5,7,13]. Failing to do so may hinder students’ ability to critically assess AI-generated information and apply it responsibly in practice [6,9,13].

Addressing this gap is crucial not only for ensuring that future orthodontists are technologically competent, but also for optimizing pedagogical efficiency and maintaining educational equity. Understanding the evidence on AI-assisted learning in orthodontics can guide educators in adopting validated tools, identify barriers to implementation, and establish best practices that align with accreditation standards and learner needs.

This systematic review and meta-analysis aims to explore and consolidate the available evidence on the use of artificial intelligence tools in orthodontic education. It seeks to identify how AI has been employed as an instructional adjunct, evaluate its effectiveness in enhancing learning outcomes such as knowledge acquisition and diagnostic skill, and understand the perceptions, attitudes, and readiness of learners and instructors toward AI adoption in academic settings.

## 2. Materials and Methods

### 2.1. Protocol and Registration

This systematic review and meta-analysis followed the PRISMA 2020 guidelines [15]. The protocol was developed a priori and structured according to both PRISMA and the Cochrane Handbook for Systematic Reviews of Interventions. The protocol was registered in PROSPERO (CRD420251067750).

### 2.2. Eligibility Criteria

Studies were included if they met the following PICOS-based criteria:

Participants (P): Orthodontic students, residents in orthodontics, or faculty members engaged in orthodontic education.

Intervention (I): Use of artificial intelligence tools as part of an educational process.

Comparisons (C): Conventional education methods, manual techniques, or no intervention (where applicable).

Outcomes (O): Changes in educational performance, knowledge acquisition, perceptions, attitudes, and self-reported or objectively assessed learning outcomes.

Study design (S): Original peer-reviewed articles, including observational studies, quasi-experimental studies, and randomized controlled trials.

### 2.3. Exclusion Criteria

Studies were excluded if they met any of the following conditions: (1) studies not focused on orthodontic education (e.g., purely clinical or diagnostic AI applications without educational context); (2) reviews, commentaries, editorials, or conference abstracts without peer review; (3) studies involving non-human subjects or preclinical simulations without student or instructor participation; and (4) duplicate publications or studies with overlapping datasets unless the most comprehensive or recent version was retained.

### 2.4. Information Sources and Search Strategy

A comprehensive literature search was performed using four major databases: PubMed (MEDLINE), Scopus, Web of Science, and Embase. The search was conducted without language restrictions and included articles published to May 2025. Additional records were identified via manual reference checks and citation tracking. The search strategy combined MeSH terms and free-text keywords related to artificial intelligence, orthodontics, and education. Example keywords included: “artificial intelligence”, “machine learning”, “deep learning”, “orthodontic education”, “dental students”, “chatbot”, “AI-assisted learning”, and “curriculum”. Boolean operators (AND, OR) were used to construct the queries. The full electronic search strategy for each database, including all search terms, Boolean operators, and date limits, is provided in Supplementary Table S1.

### 2.5. Selection Process

Two independent reviewers screened titles and abstracts. Full texts of potentially eligible studies were assessed for inclusion. Disagreements were resolved through discussion, with a third reviewer acting as arbitrator when necessary.

### 2.6. Data Collection Process

Data extraction was performed independently by two reviewers using a prepiloted data collection form. Extracted items included the study title, authors, year, country, sample characteristics, study design, AI tool description, intervention type, comparator (if any), outcomes assessed, and main findings. Discrepancies in data extraction were resolved through discussion.

### 2.7. Data Items

All studies were examined for the following variables: (1) characteristics of participants (academic level, number, country); (2) nature and purpose of the AI tool used; (3) format of intervention delivery (e.g., platform type, self-paced or instructor-led); (4) outcome measures related to student performance, perception, or acceptance; and (5) follow-up period or retention testing (if applicable).

### 2.8. Study Risk of Bias Assessment

The ROBINS-I tool was used for non-randomized studies [16]. Domains assessed included confounding, selection bias, intervention classification, measurement of outcomes, missing data, and selective reporting. Bias assessments were independently conducted by two reviewers and summarized in a tabulated format.

### 2.9. Certainty of Evidence (GRADE Assessment)

The quality of evidence for each key outcome was evaluated using the GRADE (Grading of Recommendations Assessment, Development, and Evaluation) framework [17]. Five domains were assessed: risk of bias, inconsistency, indirectness, imprecision, and publication bias. Each study was rated as high, moderate, low, or very low in overall certainty based on these factors.

### 2.10. Data Synthesis and Statistical Analysis

A meta-analysis will be considered to quantitatively integrate study results, contingent upon the availability of sufficiently reported and methodologically compatible data (e.g., standard deviations, confidence intervals, or event counts) required for deriving pooled effect estimates. The feasibility of a meta-analysis will further depend on the consistency of outcome definitions and measurement approaches. Should substantial methodological heterogeneity be detected, or if critical quantitative data are absent or reported inconsistently—compromising cross-study comparability—a meta-analysis will be deemed unsuitable. In such cases, a narrative synthesis will be employed to qualitatively summarize findings. Key trends for each outcome will be delineated, supported by summary tables and figures to enhance cross-study interpretation. This methodology aligns with PRISMA recommendations, which advocate for narrative synthesis when heterogeneity or data limitations preclude robust quantitative pooling [15].

Descriptive synthesis was performed for all outcomes across included studies. For outcomes with comparable quantitative data, such as Global Quality Scale (GQS) scores, a fixed-effects meta-analysis was conducted using inverse-variance weighting. The pooled mean and 95% confidence interval (CI) were computed. Between-study heterogeneity was assessed using the I^2^ statistic. The analysis was performed using Python (v3.11). A forest plot was generated to visually represent the individual and pooled estimates. Significance was set at *p* < 0.05.

## 3. Results

### 3.1. Study Selection Process

**Figure 1:**
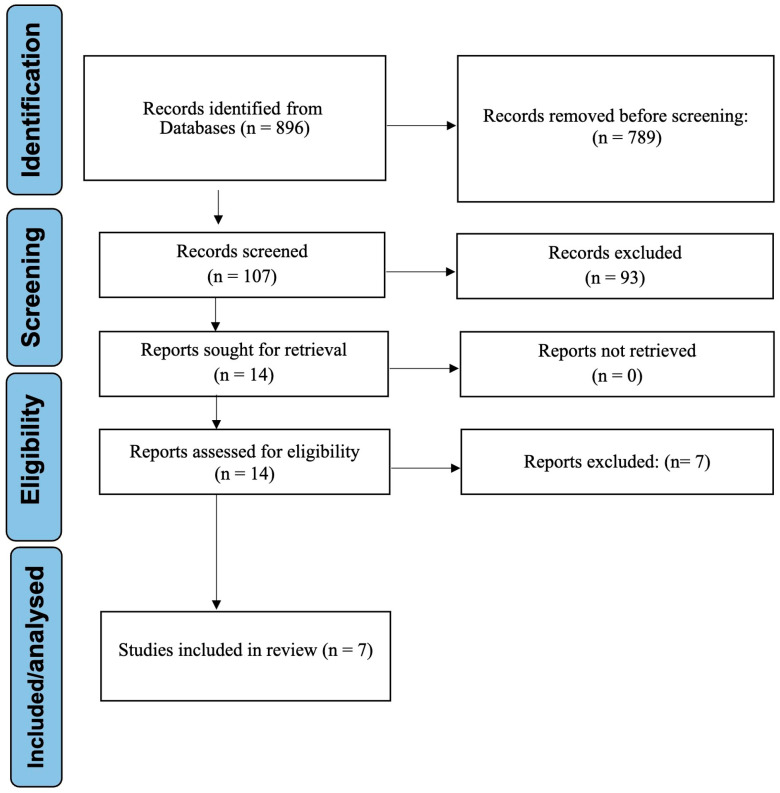
PRISMA flow diagram depicting the study selection process.

The study selection process, as detailed in the PRISMA flow diagram (Figure 1), commenced with an initial screening of 896 identified records. Following duplicate removal and the application of predefined exclusion criteria—particularly the exclusion of studies not pertaining to orthodontic education—107 articles were deemed eligible for full-text evaluation. After a thorough assessment, seven studies met all inclusion criteria and were included in the systematic review [5,7,10,11,14,18,19].

### 3.2. Data Synthesis

A meta-analysis was initially planned as part of this systematic review. However, significant methodological heterogeneity across most included studies precluded its application to the majority of outcomes. Common limitations included the absence of key statistical parameters (e.g., standard deviations, confidence intervals, or event counts) and inconsistent operational definitions, which impaired cross-study comparability. Nevertheless, sufficient data were available for one outcome (GQS) evaluating the educational quality of ChatGPT-generated content. For the remaining outcomes, a qualitative narrative synthesis was implemented. This approach involved structured comparison of trends across studies, supported by summary tables and visual representations.

### 3.3. Outcome Categorization

To facilitate clearer interpretation, studies were categorized based on the nature of their assessed outcomes: (1) those evaluating educational effectiveness through objective measures (e.g., skill acquisition, diagnostic accuracy), and (2) those exploring stakeholder perceptions and attitudes using subjective instruments (e.g., surveys, Likert scales). A summary table consolidating these distinctions is provided (Table 1).

### 3.4. Descriptive Characteristics of Included Studies

A total of seven studies were included in this systematic review, encompassing diverse methodological designs and conducted across multiple geographic regions (Table 2). Collectively, these studies involved 1101 participants, including 449 orthodontic students, 408 orthodontists, 149 faculty members, 41 program directors, and 30 general dentists. In addition, 24 trainees were included in one quasi-experimental study. The AI applications evaluated ranged from digital cephalometry and chatbot-assisted learning to surveys on stakeholder perceptions and institutional readiness. The majority employed cross-sectional surveys or quasi-experimental designs to assess orthodontic students’ and clinicians’ knowledge, perception, or performance using AI technologies. Sample sizes ranged from 24 to 298 participants, and AI applications varied across studies—from cephalometric analysis platforms and landmarking software to chatbot comparisons and in silico decision-support tools.

### 3.5. AI as an Instructional Adjunct in Orthodontic Education

All included studies explored the use of AI as a direct instructional tool in preclinical and clinical orthodontic education. Lin et al. [10] and Lin et al. [18] evaluated AI-assisted digital cephalometry platforms, demonstrating that these tools enhanced students’ landmarking accuracy by providing real-time visual feedback, thereby facilitating self-directed learning. Similarly, Metin et al. [11] compared an AI chatbot with human faculty responses to orthodontic queries and found comparable levels of user satisfaction and educational engagement, suggesting AI’s potential as a supplementary teaching aid.

Kurt et al. [19] further validated AI’s instructional role by assessing ChatGPT’s performance in delivering orthodontic information across core clinical topics (clear aligners, lingual orthodontics, esthetic braces, and TMD). Dental students evaluated the content using standardized quality tools (DISCERN and GQS), consistently rating AI-generated responses as moderate-to-good quality. These findings underscore ChatGPT’s value as an accessible, AI-powered educational adjunct for dental learners.

Hanenkrath et al. [14] surveyed North American orthodontic programs and reported that 56.1% had implemented or planned to implement AI instruction, primarily for research (60.9%) and diagnosis (39.1%). However, 87.8% lacked formal AI seminars, citing barriers like limited expertise (71.4%) and curricular space (71.4%). Despite this, 68.3% of programs encouraged residents to attend AI-related continuing education, highlighting a gap between institutional readiness and practical adoption.

Faculty-specific findings revealed varying levels of awareness and adoption. Gupta et al. [7] reported that faculty members (75%) were more aware of AI than postgraduate students (62%), with academic sources such as conferences and workshops being their primary information channels. However, only a minority of senior lecturers (34%) and professors (11%) actively supported AI integration for orthognathic surgery planning, primarily due to gaps in formal training. Mengi et al. [5] further highlighted differences between academicians and clinicians, with 41.2% of academicians being “extremely aware” of AI applications in daily life compared to only 12.5% of clinicians. While academicians strongly endorsed AI’s utility in CBCT analysis (90% agreement) and orthognathic surgery decision-making (86% agreement), clinicians remained hesitant, with 62.5% not using AI for cephalometric analysis due to cost and technical barriers. Collectively, these findings indicate AI’s growing role in autonomous learning, though faculty adoption remains inconsistent, influenced by seniority, training, and institutional support.

To enhance clarity and facilitate cross-study interpretation, Table 3 summarizes the four studies that directly evaluated the use of artificial intelligence tools as instructional adjuncts in orthodontic education. These studies employed various AI technologies—ranging from cephalometric platforms to language-based chatbots—to improve student engagement, diagnostic skills, and self-directed learning.

### 3.6. Effectiveness in Enhancing Knowledge Acquisition and Diagnostic Skill

The effectiveness of AI in fostering diagnostic competence and knowledge acquisition was explored primarily through intervention-based studies. Lin et al. [10] demonstrated that AI-supported cephalometric analysis significantly improved students’ landmarking accuracy before and after training. Additionally, Metin et al. [11] revealed that chatbot-assisted clinical reasoning scored comparably to human-guided sessions in both accuracy and satisfaction. Gupta et al. [7] also noted high expectations among postgraduate residents regarding AI’s potential to support clinical decision-making, though formal training was limited.

Kurt et al. [19] evaluated ChatGPT’s reliability in answering orthodontic queries (e.g., clear aligners, temporomandibular disorders) using the DISCERN scale and Global Quality Scale (GQS). Patients rated ChatGPT’s responses highest (mean GQS: 4.27 for clear aligners; 4.40 for lingual orthodontics), while orthodontists were more critical (mean GQS: 3.43–3.67). Despite variability, ChatGPT scored >3 (good-to-excellent) across all groups, suggesting its utility for patient education, though orthodontists emphasized the need for validation.

Faculty perspectives on AI’s diagnostic utility were mixed. Gupta et al. [7] noted that 74% of faculty agreed that AI improved cephalometric analysis accuracy, yet many cited insufficient hands-on training as a limitation. Senior lecturers (34%) were more optimistic about AI’s diagnostic applications than professors (11%), possibly reflecting generational differences in technology acceptance. Mengi et al. [5] reported that both academicians (70.6%) and clinicians (75%) viewed AI as a valuable quality control tool for treatment evaluation. However, concerns persisted, with 52% of faculty expressing apprehension about AI errors in treatment planning, underscoring the need for further validation studies. Despite these promising trends, there remains a lack of longitudinal data on how AI influences faculty skill development over time.

Table 4 summarizes studies that evaluated either objective or self-reported improvements in diagnostic skills and knowledge acquisition. Most studies assessed effectiveness using pre–post comparisons or satisfaction ratings.

### 3.7. Perceptions, Attitudes, and Readiness Toward AI in Orthodontic Education

All seven studies assessed stakeholder perceptions of AI in orthodontic education, revealing moderate-to-high interest alongside significant barriers. Hanenkrath et al. [14], Mengi et al. [5], Gupta et al. [7], Kurt et al. [19], and Metin et al. [11] identified training, technical limitations, and variation in trust across user groups as key challenges to broader AI adoption. Lin et al. [10] and Lin et al. [18] indirectly contributed to this domain by demonstrating strong student engagement and self-directed learning preferences through AI-assisted cephalometry platforms.

Faculty-specific attitudes varied considerably. Gupta et al. [7] found that while 72% of faculty supported AI integration into postgraduate curricula, practical obstacles such as high costs (89.3%) and lack of technical knowledge (94.7%) hindered implementation. Mengi et al. [5] reported that academicians were more optimistic than clinicians, with 84% perceiving AI as a collaborative partner.

Metin et al. [11] further explored these perspectives by comparing user satisfaction and trust in AI-generated orthodontic responses between students, general dentists, and orthodontists. While students and general dentists showed moderate-to-high confidence in chatbot responses (mean GQS: 3.93–4.10), orthodontists were more skeptical (mean GQS: 2.87–3.23), indicating that professional experience influences perceived reliability and readiness for AI adoption. This divergence underscores the need for targeted AI education across academic and clinical levels.

Interestingly, Kurt et al. [19] revealed that while patients and dental students viewed AI-generated responses (ChatGPT) as reliable for learning, orthodontists expressed skepticism—highlighting a divergence in perceived usefulness between learners and experts.

In the Lin et al. [10] study, 10 students using digital cephalometric platforms demonstrated rapid skill acquisition and acknowledged the value of real-time feedback in enhancing learning, though formal perception surveys were not administered. Lin et al. [18] similarly emphasized improved performance and confidence through iterative training, reinforcing the role of AI in fostering readiness and skill development.

Institutional readiness also emerged as a critical factor. Hanenkrath et al. [14] and Mengi et al. [5] highlighted disparities in support structures, with academicians advocating for curriculum integration, while clinicians faced real-world constraints. These findings emphasize the need for targeted training programs, cost-effective solutions, and institutional policies to bridge the gap between enthusiasm and implementation.

To synthesize perception and readiness-related findings, Table 5 presents a comparative summary of stakeholder attitudes toward AI in orthodontic education. This includes faculty members, postgraduate students, general dentists, program directors, and orthodontic specialists, highlighting variations in enthusiasm, institutional support, and perceived barriers.

**Figure 2:**
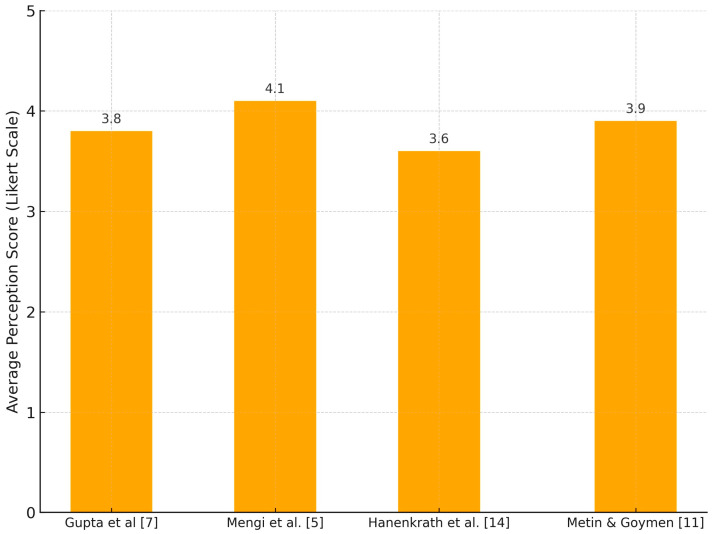
Learner perception of AI use in orthodontic education.

Figure 2 shows learner perception and attitude scores regarding AI integration in orthodontic education, based on average Likert-scale ratings from four studies. Most participants reported moderate-to-high confidence in the educational value of AI.

**Figure 3:**
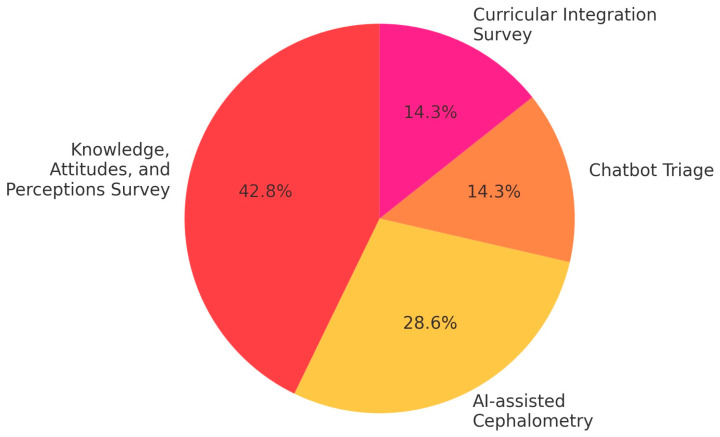
Distribution of AI applications in educational contexts.

Figure 3 displays the distribution of AI tool types and topics investigated across the included studies. Cephalometric analysis tools and perception surveys were the most common, reflecting a focus on clinical skill development and AI literacy.

### 3.8. Quantitative Synthesis of Educational Quality Scores

**Figure 4:**
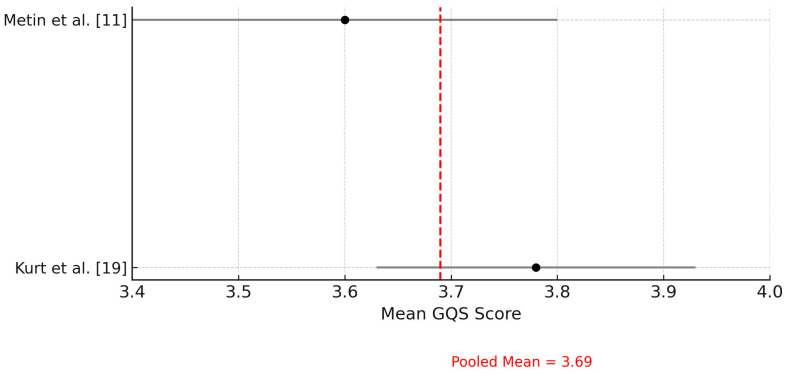
Forest plot of Global Quality Scale (GQS) scores attributed to ChatGPT across included studies. This forest plot illustrates the mean GQS scores evaluating the perceived quality of educational content generated by ChatGPT. The red dashed line represents the pooled mean GQS score, calculated using a fixed-effects model.

A meta-analysis was performed to pool the GQS scores attributed to ChatGPT in orthodontic educational content across two studies. The pooled mean GQS score was 3.69 (95% CI: 3.58 to 3.80), indicating a moderate-to-high perceived quality of information. However, heterogeneity was moderate (I^2^ = 64.5%), likely reflecting differences in evaluation context and participant profiles between studies (Figure 4).

### 3.9. Risk of Bias (ROBINS-I)

The ROBINS-I tool was applied to assess the risk of bias in non-randomized studies [16]. Three studies were rated as having a moderate risk of bias [7,10,19], three were rated as low risk [5,14,18], and one study [11] was rated as having a serious risk of bias due to lack of control over confounders and potential selection bias in participant recruitment (Table 6).

### 3.10. GRADE Evidence Summary

According to the GRADE evaluation [17], four studies were assessed as having moderate overall quality, primarily due to consistent results and precision of outcome measures [5,7,14,18]. Three studies were rated as low quality, particularly where imprecision and risk of bias were noted [10,11,19]. The most common limitations were small sample sizes and lack of randomization, which contributed to imprecision and downgrading in the quality of evidence. The summary of GRADE assessment is presented in Table 7.

## 4. Discussion

This systematic review and meta-analysis synthesized evidence from seven studies exploring the integration of AI into orthodontic education. The included studies spanned various geographic locations and methodological designs, involving a total of 1,101 participants, including orthodontic students, orthodontists, program directors, faculty, and general dentists. AI tools were used in diverse contexts—from digital cephalometric landmarking and chatbot-assisted training to stakeholder perception surveys and curriculum implementation analyses.

AI interventions, particularly in image-based diagnostic exercises, consistently enhanced students’ diagnostic accuracy and promoted autonomous learning. Lin et al. [10] demonstrated that AI-assisted cephalometric learning significantly improved landmark identification accuracy among students and fostered greater engagement compared to traditional instruction. This aligns with prior work [20], suggesting that the visual and interactive nature of AI platforms enhances spatial recognition in dental radiography.

Gupta et al. [7] further supported this by showing improved post-training scores among dental interns using AI-enhanced modules. This reflects prior reports that interactive e-learning environments driven by AI can bridge knowledge gaps and adapt to individual learning paces [21]. Faculty perspectives in Gupta’s study revealed a generational divide: younger instructors showed greater openness toward AI integration, whereas more senior educators expressed reservations—primarily due to a perceived lack of training infrastructure and curricular rigidity.

Hanenkrath et al. [14] provided an institutional perspective, reporting that only 56.1% of accredited orthodontic residency programs in North America have adopted or plan to adopt AI content. A major barrier identified was a lack of available training and curricular space (71.4%), echoing the structural limitations noted by faculty in Gupta’s study [7]. Despite this, over 60% of AI adoption in Hanenkrath’s findings was driven by research needs rather than instructional purposes [14]. This underscores the still-fragmented implementation of AI into formal educational frameworks, even when administrative support exists.

Additional support for AI’s role in clinical instruction was offered by Lin et al. [10], who emphasized the need for repeated exposure to AI-assisted modules to build student confidence. In their study, students who received three rounds of AI-supported landmarking training significantly improved their accuracy over time. These findings highlight the importance of structured, progressive exposure to AI platforms, rather than one-time interventions.

Mengi et al. [5] offered valuable insights from both academicians and clinicians. While 84% of respondents agreed that AI could enhance diagnostic quality, more than 60% of clinicians were not actively incorporating AI tools into their clinical routines. The discrepancy between belief and behavior suggests barriers beyond awareness—such as workflow integration, training time, and usability—which warrant attention in curriculum development and institutional planning.

From a clinical decision-making standpoint, Metin et al. [11] highlighted the potential of chatbot-based AI systems in orthodontic knowledge dissemination and patient education. Although ChatGPT-4 showed accuracy levels close to those of orthodontists, it lacked consistency compared to human responses. This raises concerns about the reliability of AI-generated content in critical educational scenarios. Furthermore, students and general dentists demonstrated significantly lower accuracy scores than orthodontists and top-performing chatbots, reinforcing the potential of AI tools to support foundational learning when paired with expert guidance.

Kurt et al. [19] examined AI’s value in patient and student education, and found that AI-generated responses were perceived as moderately to highly informative by students and patients, but less favorably by orthodontists. Notably, patients rated the information more positively than professionals did, reflecting a potential gap in expectations or critical standards between user groups [22]. These results reinforce the importance of integrating human oversight and verification into any AI-assisted communication tools used in educational or clinical settings [6,8]. Furthermore, the study suggested that while AI may improve accessibility to orthodontic knowledge, it cannot replace the nuanced expertise and personalized judgment offered by experienced professionals.

These perceptual differences may be rooted in varying levels of trust, digital literacy, and familiarity with AI systems. Students and patients, often newer to clinical frameworks or reliant on accessible explanations, may view AI-generated content as sufficient or even novel, whereas experienced orthodontists may apply stricter epistemological or professional standards when judging information accuracy and utility. Ethical concerns, such as accountability, transparency, and fear of diminished clinical authority, may also contribute to expert skepticism. These dynamics underscore the need for differentiated educational strategies: for example, training programs for students should include guidance on evaluating AI critically, while faculty development should address how to supervise, contextualize, and integrate AI tools without undermining professional judgment.

A recurring theme across studies was stakeholder perception. Most orthodontic students and young faculty expressed optimism regarding AI’s role in enhancing clinical education and workflow efficiency, while seasoned orthodontists and program directors were more cautious. This dichotomy suggests that broader AI adoption in orthodontics will depend on targeted faculty development programs, continuous evaluation of educational outcomes, and ethical integration strategies [6,7,8,14].

To translate these findings into actionable strategies, several implementation pathways can be considered for integrating AI into dental curricula. Elective modules focusing on AI principles and clinical applications could be introduced during preclinical years, providing foundational exposure without overwhelming core course requirements. For postgraduate learners and practicing professionals, structured continuing education programs—delivered as hybrid workshops or online certifications—may address gaps in faculty readiness and promote broader adoption. In parallel, integrated seminars or short thematic blocks embedded within existing clinical rotations can expose learners to practical AI applications, such as cephalometric analysis, diagnostic assistance, or patient communication through large language models. These phased strategies may enhance both technological fluency and pedagogical alignment while respecting curricular constraints.

From a curricular standpoint, Lin et al. [10] emphasized the lack of structured AI modules in existing orthodontic programs and recommended phased inclusion starting with elective modules followed by core integration. Aligning with this, Gupta et al. [7] advocated for hybrid learning models that combine AI tools with faculty supervision to bridge pedagogical gaps. The call for deliberate curricular scaffolding, endorsed across studies, emphasizes the importance of embedding AI literacy and critical evaluation skills early in dental training [6,23,24,25]. Such integration would not only improve technological fluency but also prepare future professionals to use AI ethically and effectively in clinical decision-making. Additionally, ethical considerations related to AI use in education require more deliberate attention. While AI tools offer promising opportunities for self-directed learning and instructional augmentation, they also introduce risks. Over-reliance on AI-generated outputs may undermine the development of independent clinical reasoning, especially if learners are not trained to critically evaluate algorithmic decisions. Furthermore, AI models may perpetuate or amplify biases present in training datasets, potentially influencing diagnostic accuracy or educational content quality. It is therefore essential to embed instruction on critical appraisal skills, transparency, and ethical AI use into any AI-enhanced curriculum to ensure safe and responsible integration.

Our meta-analysis of GQS scores further supports the narrative findings by quantitatively confirming that ChatGPT-generated orthodontic content achieves moderate levels of perceived educational quality. Despite moderate heterogeneity, the pooled results suggest consistency in ChatGPT’s performance across different evaluator groups and content domains. This quantitative reinforcement strengthens the argument for integrating ChatGPT as a supplemental tool in orthodontic education. However, the observed heterogeneity (I^2^ = 64.5%) suggests variability in participant types, evaluation contexts, or educational settings across studies, which may limit the generalizability of the pooled GQS estimate. Therefore, while the average rating indicates moderate-to-high perceived quality, this should be interpreted with caution and viewed as context-dependent.

This review is limited by the heterogeneity of the included studies in terms of sample size, educational settings, outcome measures, and types of AI tools evaluated. Although a meta-analysis was planned, quantitative synthesis was only feasible for one outcome—Global Quality Scale scores—due to the availability of standardized metrics across two studies. For all other outcomes, reliance on narrative synthesis was necessary, which may reduce the statistical power of the findings.

The predominance of self-reported data in perception-based surveys may also introduce response bias. Moreover, the small number of included studies limits generalizability and precludes subgroup analyses. Risk-of-bias and certainty-of-evidence assessments further highlighted methodological concerns in some reports. Collectively, these limitations temper the overall strength and confidence in the synthesized conclusions.

Nonetheless, this review offers several strengths. It is the first to systematically consolidate evidence on the educational applications of AI in orthodontics, drawing from diverse geographic and academic settings. The inclusion of studies targeting various stakeholder perspectives—students, faculty, clinicians, and program directors—provides a comprehensive overview of current attitudes and implementation practices. Additionally, the use of standardized appraisal tools enhances the methodological transparency and credibility of the findings. Future research should prioritize multicenter, longitudinal evaluations of AI-enhanced curricula, assess patient-related outcomes in clinical training, and explore the cost-effectiveness of AI integration in diverse educational contexts. This trend toward digital integration in orthodontics is further reflected in recent systematic reviews assessing the clinical predictability of software-based planning tools such as ClinCheck, which, while focused on treatment outcomes, underscore the increasing relevance of digitally guided interventions in both practice and pedagogy [26].

In addition, future studies should aim to evaluate the effectiveness of specific faculty training models—such as blended learning modules, peer mentoring, or simulation-based workshops—in improving technological fluency and instructional confidence. Comparative research on different curricular frameworks for AI literacy, including elective versus integrated models, would provide valuable evidence to guide academic institutions. Developing and validating standardized teaching tools, outcome measures, and accreditation guidelines for AI use in orthodontic education will also be essential to support widespread and consistent adoption.

## 5. Conclusions

AI is progressively emerging as a supportive adjunct in orthodontic education, with promising outcomes in diagnostic training, engagement, and personalized learning. The quantitative synthesis of GQS scores further validates the educational quality of ChatGPT, suggesting it may serve as a viable support tool in academic settings. While students and early-career faculty show strong enthusiasm, the full-scale integration of AI remains constrained by curricular, logistical, and perceptual barriers.

To support responsible and effective integration, institutions should consider developing formal implementation guidelines that define AI’s educational scope, ethical boundaries, and evidence-based use cases. Faculty training initiatives—focusing on both technological competence and critical appraisal—will be essential to overcome resistance and ensure consistency in delivery. Additionally, evaluating the cost-effectiveness of AI-assisted programs through pilot implementation and outcome tracking may guide scalable adoption strategies across diverse educational settings.

## Figures and Tables

**Table 1:** Summary of outcomes, measures, and key findings by study.

Study (Author, Year)	Outcome Type	Tools/Measures Used	Objective vs. Subjective	Key Findings
Lin et al. (2025) [10]	Skill improvement	Landmarking accuracy tests	Objective	Improved cephalometric accuracy
Hanenkrath et al. (2025) [14]	Curricular readiness	Survey among directors	Subjective	Limited integration, lack of training
Gupta et al. (2025) [7]	Perceptions, attitudes	Online survey (Likert)	Subjective	High expectations, limited training
Metin et al. (2025) [11]	Knowledge retention and perception	AI chatbot, comparison tests, GQS	Both	Comparable satisfaction and diagnostic performance
Mengi et al. (2024) [5]	Faculty/student attitudes	Questionnaire	Subjective	Academicians more favorable than clinicians
Kurt et al. (2024) [19]	Perceived quality	ChatGPT evaluations, DISCERN, GQS	Subjective	Patients rated highest; experts most critical
Lin et al. (2023) [18]	Skill improvement	Pre/post cephalometric training	Objective	Significant increase in diagnostic accuracy

**Table 2:** Descriptive characteristics of included studies.

Author (Year)	Country	Study Design	Participants	AI Application
Lin et al. (2025) [10]	China	Quasi-experimental	40 students, 24 trainees	Digital and AI-assisted cephalometry
Hanenkrath et al. (2025) [14]	USA/Canada	Cross-sectional	41 program directors	AI integration in curricula (research, diagnosis)
Gupta et al. (2025) [7]	India	Cross-sectional	117 postgraduate students and 149 faculty members	Knowledge, attitude, and perception (KAP)
Metin et al. (2025) [11]	Turkey	Cross-sectional	30 dentists, 30 students, 30 orthodontists	Chatbot vs. human response comparison
Mengi et al. (2024) [5]	India	Cross-sectional	50 orthodontists (academicians and clinicians) and 50 postgraduate students	Knowledge and perception on AI
Kurt et al. (2024) [19]	Turkey	Cross-sectional	30 students, 30 orthodontists	Knowledge and perception on AI
Lin et al. (2023) [18]	China	Quasi-experimental	182 orthodontic students, 298 orthodontists	AI tool in landmark training

**Table 3:** Summary of studies evaluating AI as an instructional adjunct in orthodontic education.

Study	AI Tool	Educational Use Context	Participant Group	Key Findings on Instructional Role
Lin et al. [10]	AI-assisted cephalometry	Landmarking accuracy with real-time feedback	Students, trainees	Improved accuracy and autonomous learning
Metin et al. [11]	Chatbot vs. human tutor	Query-based learning and clinical reasoning	Students, clinicians	Comparable satisfaction and performance
Kurt et al. [19]	ChatGPT (AI chatbot)	Response accuracy on clinical topics	Students, orthodontists	Rated moderate-to-good quality; educational utility
Lin et al. [18]	Digital cephalometry training	Iterative training cycles with feedback	Orthodontic students	Pre/post skill enhancement and increased confidence

**Table 4:** Summary of AI impact on knowledge and diagnostic skill.

Study	AI Tool	Objective Performance Measured	Outcome Summary
Lin et al. [10]	AI-assisted cephalometry	Yes	Improved landmarking accuracy.
Gupta et al. [7]	Perceptions on AI utility	No direct measure	High expectations for decision support. 74% faculty agreed on AI for cephalometrics; limited training.
Metin et al. [11]	Chatbot vs. Human Tutor	Yes	Comparable performance.
Mengi et al. [5]	CBCT analysis tools	Yes	90% faculty endorsed AI for complex diagnostics.
Kurt et al. [19]	ChatGPT	Yes	Patients rated AI responses highest (GQS > 4); orthodontists more critical.
Lin et al. [18]	Digital cephalometric training	Yes	Significant pre/post accuracy gain.

**Table 5:** Summary of perception and readiness toward AI.

Study	Stakeholder Group	Key Findings
Lin et al. [10]	Clinical Students	AI tools improved skills and engagement; readiness inferred via improved autonomy.
Hanenkrath et al. [14]	Program Directors	AI integration planned in 56% of programs, but 87.8% lacked structured seminars.
Gupta et al. [7]	Faculty and Postgraduates	Favorable attitudes (72%), hindered by cost and lack of technical training.
Metin et al. [11]	Students, General Dentists, Orthodontists	Students and general dentists showed moderate-to-high confidence in chatbot responses; orthodontists more skeptical.
Mengi et al. [5]	Academicians and Clinicians	Academicians supportive (84%), clinicians less engaged due to workflow barriers.
Kurt et al. [19]	Students, Patients, Experts	Patients rated AI responses highly; orthodontists more critical; dental students moderate.
Lin et al. [18]	Orthodontic Students	Repeated AI-assisted training increased confidence and accuracy; readiness inferred.

**Table 6:** Risk-of-bias assessment (ROBINS-I).

Study	Bias due to Confounding	Bias in Selection of Participants	Bias in Classification of Interventions	Bias Due to Deviations from Intended Interventions	Bias due to Missing Data	Bias in Measurement of Outcomes	Bias in Selection of Reported Result	Overall Risk of Bias
Lin et al. [10]	Moderate	Low	Low	Low	Low	Low	Low	Moderate
Gupta et al. [7]	Moderate	Moderate	Low	Low	Low	Low	Low	Moderate
Metin et al. [11]	Serious	Serious	Moderate	Moderate	Low	Serious	Low	Serious
Hanenkrath et al. [14]	Low	Low	Low	Low	Low	Low	Low	Low
Mengi et al. [5]	Low	Low	Low	Low	Low	Low	Low	Low
Kurt et al. [19]	Moderate	Moderate	Low	Low	Low	Low	Low	Moderate
Lin et al. [18]	Low	Low	Low	Low	Low	Low	Low	Low

**Table 7:** GRADE evidence profile.

Study	Risk of Bias	Inconsistency	Indirectness	Imprecision	Publication Bias	Overall Quality
Lin et al. [10]	Moderate	Not Serious	Not Serious	Serious	Undetected	Low
Gupta et al. [7]	Moderate	Not Serious	Not Serious	Not Serious	Undetected	Moderate
Metin et al. [11]	Serious	Not Serious	Not Serious	Serious	Undetected	Low
Hanenkrath et al. [14]	Low	Not Serious	Not Serious	Not Serious	Undetected	Moderate
Mengi et al. [5]	Low	Not Serious	Not Serious	Not Serious	Undetected	Moderate
Kurt et al. [19]	Moderate	Serious	Not Serious	Serious	Undetected	Low
Lin et al. [18]	Low	Not Serious	Not Serious	Not Serious	Undetected	Moderate

## Data Availability

The datasets used and/or analyzed during the current study are available from the corresponding author upon reasonable request.

## Supplementary Materials

The following supporting information can be downloaded at: https://www.mdpi.com/article/10.3390/jcm14155487/s1, Table S1. Detailed Search Strategies Used for Each Database
